# Evaluating quality of life in third molar surgery: a scoping review of the postoperative symptom severity (PoSSe) scale

**DOI:** 10.4317/medoral.26839

**Published:** 2025-02-15

**Authors:** Eduardo Frederico Eduardo Maferano, Edson Luiz Cetira Filho, Paulo Goberlânio de Barros Silva, Ana Flávia Granville-Garcia, Ramon Targino Firmino, Matheus de França Perazzo, Paulo Ricardo Martins-Filho, Fábio Wildson Gurgel Costa

**Affiliations:** 1 DDS, MSc, PhD student. Department of Dentistry, Faculty of Health Sciences, Zambeze University, Tete City, Mozambique; 2 DDS, MSc, PhD. Postgraduate Program in Dentistry Christus University Center, Ceará, Fortaleza, Brazil; 3 DDS, MSc, PhD. Division of Oral Pathology, School of Dentistry, Christus University Center, Fortaleza, Ceará, Brazil; 4 DDS, MSc, PhD. State University of Paraíba, Department of Dentistry, Campina Grande, Paraíba, Brazil.; 5 DDS, MSc, PhD. Federal University of Campina Grande, Academic Unit of Biological Sciences, Patos, Paraíba, Brazil; 6 DDS, MSc, PhD. Federal University of Goiás, Department of Dentistry, Goiânia, Brazil; 7 DDS, MSc, PhD. Investigative Pathology Laboratory, Federal University of Sergipe, Sergipe, Aracaju, Brazil; 8 DDS, MSc, PhD. Postgraduate Program in Dentistry, Faculty of Dentistry, Federal University of Ceará, Ceará, Fortaleza, Brazil

## Abstract

**Background:**

This scoping review evaluated the key dimensions of quality of life impacted by third molar surgery as assessed by the Postoperative Symptom Severity (PoSSe) scale, and their variations across diverse populations and clinical contexts.

**Material and Methods:**

A comprehensive literature search was performed across multiple databases including MEDLINE, EMBASE, CINAHL, PsycINFO, The Cochrane Library, Livivo, LILACS, Web of Science, Scopus, Epistemonikos, and Google Scholar on April 30, 2024, with an update on July 31, 2024. We included analytical observational studies and randomized clinical trials that utilized the PoSSe scale to assess quality of life. There were no restrictions based on language, location, or publication period. Data from eligible studies were extracted and analyzed descriptively.

**Results:**

The search identified 3,438 records, with 31 studies ultimately included. These studies employed the PoSSe scale in various methodological designs to primarily assess quality of life following lower third molar removal. The dimensions most affected were feeding, appearance, and pain, which showed significant correlations with edema, trismus, and analgesic use. Additional influencing factors included gender, tobacco use, surgeon skill level, Pell and Gregory classification, and preemptive analgesia.

**Conclusions:**

This review has demonstrated the PoSSe scale's effectiveness in evaluating the multifaceted impacts of third molar surgery on patient quality of life, sensitive to differences in surgical techniques, surgeon experience, and patient-specific factors. Future research should explore longitudinal assessments with the PoSSe scale to optimize surgical practices and improve long-term patient outcomes.

** Key words:**Tooth extraction, quality of life, third molar, scoping review.

## Introduction

The surgical extraction of mandibular third molars under local anesthesia is a routine procedure in oral and maxillofacial surgery, primarily due to the frequent incidence of impaction. While most individuals develop third molars, a considerable number present with at least one impacted tooth. The high occurrence of impaction is attributed to both genetic predispositions and environmental influences (1-3).

Indications for third molar extraction include caries (in partially erupted third molars and/or adjacent second molars), periapical pathology, recurrent pericoronitis, infection such as abscess or osteomyelitis, internal and/or external root resorption (of the third molar or adjacent tooth), mandibular angle fracture, trauma and tooth fracture, extraction for dental autotransplantation, orthodontic reasons, and periodontal disease (3).

The surgical procedure involves reflecting the mucoperiosteal flap, with or without bone removal. Postoperative complications can include pain, edema, trismus, reduced masticatory function, alveolitis, and neurological complications. Pain and swelling are triggered by an acute inflammatory response in the surgical area, characterized by vasodilation and the influx of pro-inflammatory mediators, significantly impacting the quality of life and well-being in the postoperative period (2,3).

Health-Related Quality of Life (HRQoL) is a multidimensional concept that encompasses an individual’s functioning in daily life and their perception in physical, mental, and social domains. HRQoL is influenced by the presence of a disease or the treatment received (4,5), and is often assessed in cohort studies and randomized clinical trials using patient self-administered questionnaires. These outcomes, reflecting the patients' perspective, frequently differ from clinical or objective assessments (5). This construct, when applied to the dental context, is referred to as Oral Health-Related Quality of Life (OHRQoL).

Several instruments have been developed to assess Oral Health-Related Quality of Life (OHRQoL). These tools are designed to evaluate the functional, psychological, and social impacts of diseases and disorders that affect the oral cavity and its associated structures. NoTable examples include the 14-item Oral Health Impact Profile (OHIP-14), the Oral Impacts on Daily Performances (OIDP), and the Postoperative Symptom Severity (PoSSe) scale (4-6).

The Postoperative Symptom Severity (PoSSe) scale, specifically designed with questions to assess patients undergoing third molar extraction, evaluates the quality of life during the postoperative period (4). The scale comprises 15 items across seven subscales addressing the main adverse effects reported by patients’ post-surgery, such as eating, oral and vocal function, appearance, physical discomfort, and social interaction, typically in the first week after the procedure. Recognized for its reliability and sensitivity, the PoSSe scale effectively measures the clinical impact of postoperative discomfort and accurately reflects patients' perceptions of symptom severity after lower third molar extraction (4).

In recent years, the PoSSe scale has been widely adopted in a range of international studies, including observational and clinical trials. These investigations explore a variety of demographic and surgical variables, including the relationship between the duration of surgery and the severity of postoperative symptoms, the effectiveness of varying doses, routes, and timing of corticosteroid administration, the types of anesthetics used during surgery, the surgical expertise of professionals, and the comparative effectiveness of different anti-inflammatory medications. This widespread usage underscores the scientific community's interest in understanding and quantifying the impact of symptoms and discomforts associated with third molar surgery in the postoperative period (1,3,7-11), enhancing the scope of patient-centered care in dental practice.

An initial search in the MEDLINE, Cochrane Database of Systematic Reviews, and JBI Evidence Synthesis databases found no ongoing or recent scoping reviews on the topic. Therefore, this scoping review aimed to analyze the quality of life after third molar surgery using the PoSSe scale in observational studies and clinical trials.

## Material and Methods

This scoping review adhered to the PRISMA-ScR (Preferred Reporting Items for Systematic Reviews and Meta-Analyses Extension for Scoping Reviews) checklist (12). The protocol for this study was registered with the Open Science Framework (https://osf.io/) under the DOI: 10.17605/OSF.IO/KM93W.

- Identification of Research Questions

The research questions for this scoping review are as follows:

1. What are the main dimensions of quality of life assessed by the PoSSe scale after third molar surgery, and how do these vary among different patient populations or clinical contexts?

2. What evidence exists regarding the reliability and validity of the PoSSe scale in evaluating the quality of life outcomes specifically related to third molar surgery?

3. Which aspects of quality of life are predominantly affected by third molar surgery, as evaluated by the PoSSe scale, and what are the contributing factors to these outcomes?

- Search Strategy

The search strategy was designed to locate both published and unpublished studies using a three-step approach:

1. First Step: Conducted in databases including MEDLINE (PubMed), Excerpta Medica database (EMBASE), Cumulative Index to Nursing and Allied Health Literature (CINAHL), Psychological Information Database (PsycINFO), The Cochrane Library, Livivo, LILACS, Web of Science, Scopus, Epistemonikos, and Google Scholar (for grey literature). Keywords from titles and abstracts of relevant articles, as well as indexing terms, were utilized to develop a comprehensive search strategy. In PubMed, the following descriptors were employed: "molar, third", "Quality of Life", and "postoperative symptom severity scale," along with their synonyms. The descriptors were combined using the Boolean operator AND, and the synonyms were combined using the Boolean operator OR. This search strategy was adapted for the different databases (details in Table 1).

2. Second Step: This search was executed in the aforementioned databases on April 25, 2024.

3. Third Step: The search was updated on July 31, 2024, to include new publications.

- Eligibility Criteria

This scoping review included analytical observational studies such as prospective and retrospective cohort studies, case-control studies, analytical cross-sectional studies, and both randomized and non-randomized clinical trials. These studies were conducted in surgical centers or oral and maxillofacial surgery clinics and adhered to recommended practices for conducting surgery. Studies included evaluated the quality of life of patients undergoing third molar surgery using the PoSSe scale. There were no restrictions regarding language, location, or publication period. Exclusions were applied to book chapters, literature reviews, studies involving pregnant or lactating patients, patients with pre-existing cognitive impairments, preoperative inflammatory or infectious conditions, chronic systemic diseases, studies that utilized the PoSSe scale before its validation, and studies excluded due to the lack of indexing in PubMed-Medline.

- Study Screening and Selection

**Figure 1:**
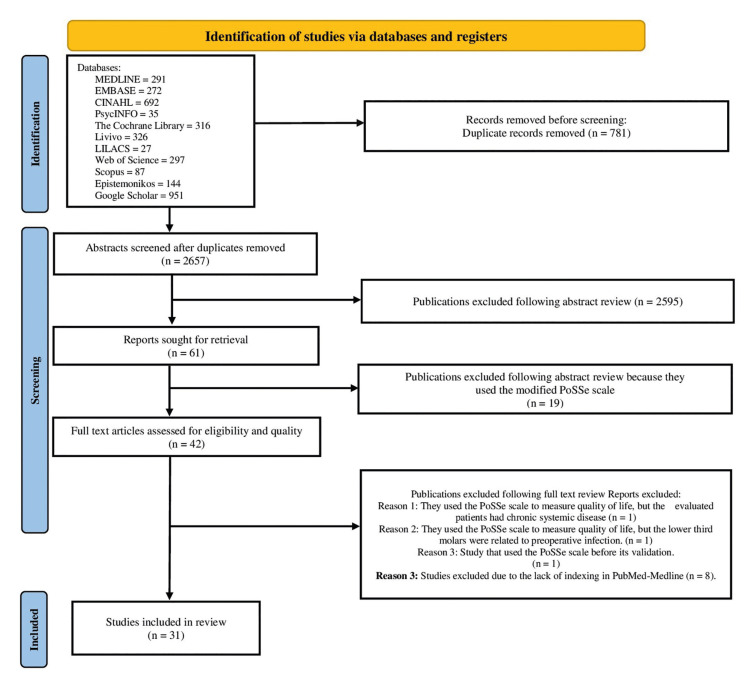
PRISMA flowchart.

Following the retrieval of the literature, studies were exported to the Rayyan application, where duplicates were removed. Two researchers (EFEM and ECF), independently screened the titles and abstracts against the eligibility criteria and selected articles for the scoping review. A third researcher (FWGC), an expert in scoping reviews and the subject area, verified these selections and resolved any discrepancies. The complete screening and selection process is outlined in the PRISMA flowchart (Fig. 1).

- Main Outcome

The primary outcome of this review was the OHRQoL following third molar extraction, using the PoSSe scale. The PoSSe scale measures adverse effects across seven subscales: eating, speech, sensation, appearance, pain, discomfort, and interference with daily activities. Responses to each question are scored and summed to produce a percentage, where the most severe response scores 100%, and the least severe scores 0% (4).

- Data Extraction

Data extraction was independently performed by two researchers (RTF and PGB) from articles that met the inclusion criteria. Extracted data included author names, publication year, study country, objectives, sample size, groups, methodological design, outcome measures, number of teeth extracted per participant, study variables, and main findings. Where data were missing, Correspondences were contacted. A third researcher (AFGG), an expert in scoping reviews, analyzed and compared the extracted data to address discrepancies. Data were organized in an Excel spreadsheet without the use of specific software for extraction and management.

- Compilation, Summary, and Presentation of Data

The compiled data pertained to the quality of life impacts measured by the PoSSe scale in patients undergoing third molar surgery. Given the nature of the scoping review, no meta-analysis was conducted. The results were synthesized narratively.

## Results

The search strategy yielded 2,487 records, along with an additional 951 from gray literature repositories. After removing 781 duplicates, 2,657 unique records underwent title and abstract screening. Of these, 61 articles were selected for full-text review. Following a thorough evaluation, 19 articles were excluded for using a modified version of the PoSSe scale. Three additional articles were excluded based on the established eligibility criteria: one applied the PoSSe scale to patients with chronic systemic diseases, another involved lower third molars associated with preoperative infections, and one study utilized the PoSSe scale prior to its validation. Of the remaining 39 articles, eight were excluded due to the lack of indexing in PubMed-Medline (Supplement 1), and - 31 articles (1-4,7-11,13-34) met the inclusion criteria and were analyzed, as illustrated in Fig. 1.

- Study Characteristics

The included studies employed a range of methodological designs. There were 23 randomized clinical trials (1,2,7-11,13,14,16,19,20-27), including six single-blind (1,7,21,24,27,34), six double-blind (10,13,16,19,22,25), one non-blind randomized split-mouth trial (20); and ten randomized clinical trials (2,8,9,11,14,23,26,29,31,33) that did not specify blinding. Additionally, the review included seven non-randomized clinical trials (17,18,28,30,32), one cross-sectional study (4) and two retrospective observational studies (3,15).

The geographical distribution of these studies included China (*n*=8) (3,13,15,24,28,31,33,34), Italy (*n*=4) (1,9,18,30), India (*n*=3) (7,10,17), the United Kingdom (*n*=2) (8,16), Ireland (*n*=2) (19,32), Syria (*n*=1) (2), the United States (*n*=2) (21,27), Israel (*n*=1) (22), Turkey (*n*=2) (26,29), Nepal (*n*=1) (11), Peru (*n*=1) (14), Scotland (*n*=1) (4), Bologna (*n*=1) (20), Brazil (*n*=1) (23), and Spain (*n*=1) (25), as detailed in Supplement 2.

Some of the included studies used the PoSSe scale as the primary outcome measure to assess postoperative quality of life (3,4,10,13,16,19,20,22,26,28). Other studies evaluated postoperative quality of life using the PoSSe scale as a secondary outcome measure (1,2,7,8,9,10,11,15,17,18). Among the 31 analyzed, 29 focused on the quality of life following the surgical removal of impacted lower third molars (1-4,7,8,10,11,13-31,33,34). Two articles specifically examined the quality of life after the removal of both upper and lower third molars (9,32).

Among the 31 included articles, the majority (n = 12) used the PoSSe scale to evaluate postoperative quality of life using following the removal of a single lower third molar (1,7,8,13,14,16-19,28,30,33). Nine studies assessed quality of life after bilateral removal of lower third molars (2,9,10,20-23,26,34). Additionally, thirteen studies did not specify the number of third molars removed (3,4,11,15,24,25,27,29,31,32). Details and characteristics of these studies are comprehensively documented in Supplement 2.

In the following sections, we synthesize the main dimensions of quality of life assessed by the PoSSe scale after third molar extraction surgery and examine how these vary across different clinical contexts. We also evaluate the existing evidence regarding the reliability and validity of the PoSSe scale in assessing specific quality of life outcomes related to third molar extraction, highlighting the key aspects of quality of life predominantly affected by third molar extraction as per the PoSSe scale and the contributing factors to these outcomes.

- Main Dimensions of Quality of Life Evaluated by the PoSSe Scale After Third Molar Extraction Surgery and Their Variation Across Different Clinical Contexts

Most studies found statistically significant differences in the mean total PoSSe scores across groups (3,4,8-10,13,14,18,20-23,28). However, some studies found statistically significant differences only in specific PoSSe subscale scores, such as those for eating (7,11,16,17,33), speech (20,33), appearance (1,7,11,17,20,30,33), pain (1,2,16,17,33), illness (11,16), and interference with daily activities (4,17,19,20,23,33). Conversely, other studies did not find differences in total mean PoSSE scores between groups or outcomes (24,25,26,29,31,32,34).

Significant correlations were observed between the PoSSe scores for appearance, pain, and eating, and clinical outcomes such as edema, the consumption of analgesics postoperatively, and mouth opening (trismus) (1,4,18). In addition, the eating and interference with daily activities subscales showed higher correlations with the overall PoSSe score (4,18).

- Contributing Factors to the Mean Total PoSSe Score for Patients After Third Molar Surgery

The mean total PoSSe score following third molar surgery was impacted by a range of clinical factors. Significant among these is the surgical expertise of the dentists performing third molar extractions under local anesthesia. Additional contributing factors included gender, tobacco use, anatomical considerations such as the relationship of the molar to the ramus and available space, preoperative symptoms, and the use of preoperative antibiotic prophylaxis. The methods employed, specifically osteotomy and odontectomy during mandibular third molar surgery, also play a role, alongside the duration of the procedure, and the number of impacted teeth. Other factors included preoperative dental anxiety, the preemptive and preventive use of corticosteroids, the Pell-Gregory classification, corticosteroid administration during surgery, the application of concentrated growth factors post-surgery, and various postoperative therapies aimed at controlling inflammation (3,4,8,9,18,21,23,28,29).

## Discussion

This scoping review critically evaluates the impact of various clinical and demographic factors on the PoSSe scale scores following third molar surgery. By assessing these diverse influences, we aim to elucidate the nuances of how surgical techniques, patient characteristics, and therapeutic interventions collectively affect postoperative quality of life.

The surgeon's experience plays a crucial role in postoperative outcomes. Patients undergoing third molar surgery under local anesthesia by more experienced professionals often report better postoperative quality of life, as reflected in lower mean total scores on the PoSSe scale (8). Additionally, the duration of surgery under local anesthesia is directly correlated with PoSSe scores, with longer procedures generally leading to worse outcomes for patients, highlighting the importance of efficient surgical techniques (3). Different types of surgery may have varied postoperative impacts on patients' perceptions of the procedure and their quality of life. For example, the use of advanced surgical instruments, such as piezoelectric devices, has been shown to significantly improve postoperative outcomes by reducing PoSSe scores, suggesting that technological advancements in surgical tools can enhance patients' perceptions of the impact of surgery during the postoperative period (17,20,21).

Gender differences have emerged as a significant factor, with women generally experiencing worse outcomes than men across most subscales, except for pain and sensation (20). This highlights the need for tailored approaches in managing postoperative care to address gender-specific responses to surgery. Additionally, factors such as tobacco use and the number of impacted teeth also play crucial roles in influencing the PoSSe scores. Particularly, teeth classified under Pell and Gregory Classes II and III, which are more complex surgical cases, are associated with higher PoSSe scores and therefore worse postoperative discomfort (4,18).

Various therapeutic interventions have been beneficial in managing postoperative inflammation and improving quality of life. Techniques such as submucosal dexamethasone injections and the use of elastic therapeutic bandaging and cryotherapy are linked with lower PoSSe scores (9,14,22). This suggests that proactive anti-inflammatory management is crucial for enhancing patient outcomes.

The use of long-acting anesthetics, such as 0.75% ropivacaine, during third molar removal offers significant advantages for patients' quality of life, particularly in postoperative pain management. Compared to other anesthetics, ropivacaine provides longer-lasting anesthesia, resulting in an extended period of postoperative analgesia. Ropivacaine use is associated with lower scores on the Postoperative Symptom Severity (PoSSe) scale, indicating reduced discomfort in subscales such as pain, eating, and interference with daily activities.

Objective measures such as facial edema and the severity of trismus correlate strongly with specific PoSSe subscales. The appearance and pain subscales, for instance, show significant variations corresponding to the degree of facial swelling and analgesic consumption, respectively (1,18). This connection between objective clinical measures and subjective patient experiences provides a comprehensive view of surgical impact and recovery.

When selecting the surgical technique for third molar extraction, it is essential to prioritize more conservative approaches. Coronectomy is a safe and effective option, particularly in cases where the dental roots are in close proximity to the inferior alveolar nerve. By preserving the roots and removing only the crown, this technique significantly reduces the risk of injury to the inferior alveolar nerve, which is commonly associated with complete extraction, while also promoting better postoperative outcomes. As a less invasive procedure, coronectomy minimizes surgical trauma, reduces the risk of complications such as mandibular fractures and paresthesias, and shortens operative time, thereby improving the patient's quality of life during the recovery period (3,35).

This scoping review presents several limitations that should be considered. A significant limitation is that most of the studies analyzed conducted a cross-sectional assessment of quality of life using the PoSSe scale, meaning evaluations were made at a single point in time. This approach limits the understanding of patient evolution during the postoperative period, as it does not capture variations in quality of life over time.

Additionally, the heterogeneity of the included studies may compromise the comparability of results, as different methodologies and patient populations were used. There is also variation in how PoSSe scale scores were reported; some studies evaluated both the total score and subscales, while others focused solely on the total score, making it difficult to draw definitive conclusions about the overall impact of certain factors. Furthermore, some of the included studies relied exclusively on subjective patient reports without incorporating objective clinical measures, which may reduce the reliability of the findings. Finally, the review did not account for all potential confounding factors, such as comorbidities and socioeconomic status, which may also influence postoperative quality of life.

This scoping review presents several strengths: it is the first review focused on the PoSSe scale, offering a new perspective on the assessment of oral health-related quality of life in the context of third molar surgery. Additionally, the review encompasses a variety of clinical and demographic factors that influence the PoSSe scale scores, providing a detailed and multifaceted view of the impact of surgery on patients' quality of life.

Conclusion

This review underscores the effectiveness of the PoSSe scale in assessing the impacts of third molar surgery on patient quality of life. Key findings indicate that the scale effectively captures variations in surgical techniques, surgeon experience, and patient-specific factors. Future research should focus on longitudinal assessments using the PoSSe scale to improve surgical practices and patient care. Additionally, considering a broader range of confounding factors, including comorbidities and socioeconomic status, will help clarify their influence on postoperative quality of life.

## Figures and Tables

**Table 1:** Search strategy of the different databases used in the study.

Data base	Search strategy
MEDLINE (PubMed)	("molar, third"[MeSH Terms] OR "molar third"[All Fields] OR "third molar"[All Fields] OR "third molars"[All Fields] OR "third molar"[All Fields] OR "third molars"[All Fields] OR "Wisdom Tooth"[All Fields] OR "Wisdom Teeth"[All Fields]) AND ("Quality of Life"[MeSH Terms] OR "Quality of Life"[All Fields] OR "Life quality"[All Fields] OR "Life qualities"[All Fields] OR "health related quality of life"[All Fields] OR "health related quality of life"[All Fields] OR "QOL"[All Fields] OR "HRQOL"[All Fields] OR "Health status"[All Fields] OR "Living quality"[All Fields] OR "postoperative symptom severity scale"[All Fields] OR "PoSSe scale"[All Fields] OR "POSSE"[All Fields])
EMBASE (Medica dataBASE)	(‘third, molar' OR ‘third molar'/exp OR ‘third molar' OR ‘third molars' OR ‘third-molar' OR ‘third-molars' OR ‘wisdom tooth' OR ‘wisdom teeth') AND (‘quality of life'/exp OR ‘quality of life' OR ‘quality of life assessment' OR ‘quality of life index' OR ‘health-related quality of life' OR ‘health related quality of life' OR ‘qol' OR ‘hrqol' OR ‘health status' OR ‘living quality' OR ‘postoperative symptom severity scale' OR ‘posse scale' OR ‘posse') AND [embase]/lim
CINAHL	( MH "THIRD molars" OR "Molar, Third" OR "Third Molar" OR "Third Molars" OR "Third-molar" OR "Third-molars" OR "Wisdom Tooth" OR "Wisdom Teeth" ) AND ( MH "Quality of Life" OR "Quality of Life" OR "Life quality" OR "Life qualities" OR "Health-related quality of life" OR "Health related quality of life" OR "QOL" OR "HRQOL" OR "Health status" OR "Living quality" OR "postoperative symptom severity scale" OR "PoSSe scale" OR "POSSE" )
Psychological Information Database (PsycINFO)	Any Field: Molar, Third OR Any Field: Third Molar OR Any Field: Third Molars OR Any Field: Third-molar OR Any Field: Third-molars OR Any Field: Wisdom Tooth OR Any Field: Wisdom Teeth AND Any Field: Quality of Life OR Any Field: Life quality OR Any Field: Life qualities OR Any Field: Health-related quality of life OR Any Field: Health related quality of life OR Any Field: QOL OR Any Field: HRQOL OR Any Field: Health status OR Any Field: Living quality OR Any Field: postoperative symptom severity scale OR Any Field: PoSSe scale OR Any Field: POSSE
The Cochrane Library	((Molar, Third OR Third Molar OR Third Molars OR Third-molar OR Third-molars OR Wisdom Tooth OR Wisdom Teeth) AND (Quality of Life OR Life quality OR Life qualities OR Health-related quality of life OR Health related quality of life OR QOL OR HRQOL OR Health status OR Living quality OR postoperative symptom severity scale OR PoSSe scale OR POSSE))
Livivo	("Molar, Third" OR "Third Molar" OR "Third Molars" OR "Third-molar" OR "Third-molars" OR "Wisdom Tooth" OR "Wisdom Teeth") AND ("Quality of Life" OR "Life quality" OR "Life qualities" OR "Health-related quality of life" OR "Health related quality of life" OR "QOL" OR "HRQOL" OR "Health status" OR "Living quality" OR "postoperative symptom severity scale" OR "PoSSe scale" OR "POSSE")
LILACS (BVS)	((molar, third) OR (third molar) OR (third molars) OR (third-molar) OR (third-molars) OR (wisdom tooth) OR (wisdom teeth)) AND ((quality of life) OR (life quality) OR (life qualities) OR (health-related quality of life) OR (health related quality of life) OR (qol) OR (hrqol) OR (health status) OR (living quality) OR (postoperative symptom severity scale) OR (posse scale) OR (posse)) AND ( db:("LILACS"))
Web of Science	(ALL=("Molar, Third" OR "Third Molar" OR "Third Molars" OR "Third-molar" OR "Third-molars" OR "Wisdom Tooth" OR "Wisdom Teeth")) AND ALL=("Quality of Life" OR "Life quality" OR "Life qualities" OR "Health-related quality of life" OR "Health related quality of life" OR "QOL" OR "HRQOL" OR "Health status" OR "Living quality" OR "postoperative symptom severity scale" OR "PoSSe scale" OR "POSSE")
Scopus	( ALL ( ‘molar, AND third' OR ‘third AND molar' OR ‘third AND molars' OR ‘third-molar' OR ‘third-molars' OR ‘wisdom AND tooth' OR ‘wisdom AND teeth' ) AND ALL ( ‘quality AND of AND life' OR ‘life AND quality' OR ‘life AND qualities' OR ‘health-related AND quality AND of AND life' OR ‘health AND related AND quality AND of AND life' OR ‘qol' OR ‘hrqol' OR ‘health AND status' OR ‘living AND quality' OR ‘postoperative AND symptom AND severity AND scale' OR ‘posse AND scale' OR ‘posse' ) )
Epistemonikos	(title:((title:("Molar, Third" OR "Third Molar" OR "Third Molars" OR "Third-molar" OR "Third-molars" OR "Wisdom Tooth" OR "Wisdom Teeth") OR abstract:("Molar, Third" OR "Third Molar" OR "Third Molars" OR "Third-molar" OR "Third-molars" OR "Wisdom Tooth" OR "Wisdom Teeth")) AND (title:("Quality of Life" OR "Life quality" OR "Life qualities" OR "Health-related quality of life" OR "Health related quality of life" OR "QOL" OR "HRQOL" OR "Health status" OR "Living quality" OR "postoperative symptom severity scale" OR "PoSSe scale" OR "POSSE") OR abstract:("Quality of Life" OR "Life quality" OR "Life qualities" OR "Health-related quality of life" OR "Health related quality of life" OR "QOL" OR "HRQOL" OR "Health status" OR "Living quality" OR "postoperative symptom severity scale" OR "PoSSe scale" OR "POSSE"))) OR abstract:((title:("Molar, Third" OR "Third Molar" OR "Third Molars" OR "Third-molar" OR "Third-molars" OR "Wisdom Tooth" OR "Wisdom Teeth") OR abstract:("Molar, Third" OR "Third Molar" OR "Third Molars" OR "Third-molar" OR "Third-molars" OR "Wisdom Tooth" OR "Wisdom Teeth")) AND (title:("Quality of Life" OR "Life quality" OR "Life qualities" OR "Health-related quality of life" OR "Health related quality of life" OR "QOL" OR "HRQOL" OR "Health status" OR "Living quality" OR "postoperative symptom severity scale" OR "PoSSe scale" OR "POSSE") OR abstract:("Quality of Life" OR "Life quality" OR "Life qualities" OR "Health-related quality of life" OR "Health related quality of life" OR "QOL" OR "HRQOL" OR "Health status" OR "Living quality" OR "postoperative symptom severity scale" OR "PoSSe scale" OR "POSSE"))))
Google Scholar (Grey literature)	"Molar, Third" OR "Third Molar" OR "Third Molars" OR "Third-molar" OR "Third-molars" OR "Wisdom Tooth" OR "Wisdom Teeth" AND "Quality of Life" OR "Life quality" OR "Life qualities" OR "Health-related quality of life" OR "Health related quality of life" OR "QOL" OR "HRQOL" OR "Health status" OR "Living quality" OR "postoperative symptom severity scale" OR "PoSSe scale" OR "POSSE"
